# Dopamine-dependent social information processing in non-human primates

**DOI:** 10.1007/s00213-018-4831-x

**Published:** 2018-01-14

**Authors:** Young-A Lee, Sarah Lionnet, Akemi Kato, Yukiori Goto

**Affiliations:** 1 Department of Food Science and Nutrition, Daegu Catholic University, Gyeongsan, Gyeongbuk 38430 South Korea; 2 Ecole Nationale Veterinaire de Toulouse, 31076 Toulouse, France; 3 Primate Research Institute, Kyoto University, 41-2 Kanrin, Inuyama, Aichi 484-8506 Japan

**Keywords:** Dopamine, Near-infrared spectroscopy, Macaques, Prefrontal cortex, Social cognition

## Abstract

**Rationale:**

Dopamine (DA) is a neurotransmitter whose roles have been suggested in various aspects of brain functions. Recent studies in rodents have reported its roles in social function. However, how DA is involved in social information processing in primates has largely remained unclear.

**Objectives:**

We investigated prefrontal cortical (PFC) activities associated with social vs. nonsocial visual stimulus processing.

**Methods:**

Near-infrared spectroscopy (NIRS) was applied to Japanese macaques, along with pharmacological manipulations of DA transmission, while they were gazing at social and nonsocial visual stimuli.

**Results:**

Oxygenated (oxy-Hb) and deoxygenated (deoxy-Hb) hemoglobin changes as well as functional connectivity based on such Hb changes within the PFC network which were distinct between social and nonsocial stimuli were observed. Administration of both D1 and D2 receptor antagonists affected the Hb changes associated with social stimuli, whereas D1, but not D2, receptor antagonist affected the Hb changes associated with nonsocial stimuli.

**Conclusions:**

These results suggest that mesocortical DA transmission in the PFC plays significant roles in social information processing, which involves both D1 and D2 receptor activation, in nonhuman primates. However, D1 and D2 receptor signaling in the PFC mediates different aspects of social vs. nonsocial information processing.

**Electronic supplementary material:**

The online version of this article (10.1007/s00213-018-4831-x) contains supplementary material, which is available to authorized users.

## Introduction

Mesocortical dopamine (DA) innervations arising from the ventral tegmental area into prefrontal cortical (PFC) regions have been shown to play crucial roles in an assortment of cognitive and affective functions, such as working memory, behavioral flexibility, and decision making (Arnsten 1997; Robbins 2000; Seamans and Yang 2004). Although mesocortical DA innervations into the PFC are somewhat anatomically distinct in terms of layer specificity between species (Berger et al. 1988; Raghanti et al. 2008; Williams and Goldman-Rakic 1993), the roles of the mesocortical DA pathways in PFC functions appear to be relatively conserved, as pharmacological manipulations of DA transmission in the PFC in at least rodents and non-human primates cause similar cognitive and affective dysfunctions.

Accumulating evidence suggests that DA transmission is also involved in social functions (Skuse and Gallagher 2009). Such studies investigating the roles of DA in social functions have mostly been conducted in rodents. In these rodent studies, genetic and pharmacological manipulations of DA transmission have been shown to alter social interactions (Corbett et al. 1993; Gunaydin et al. 2014). In contrast, social functions in primate species including humans are thought to be substantially different from those in rodents, in which primate species extensively rely on visual information for social functioning, such as recognition of facial expressions of others. In humans, although studies have shown that the extrastriate cortex is involved in face recognition (Allison et al. 1994), recognition of more complex aspects of social cues in faces such as emotional expressions and distinction between self and others depends on the PFC (Forbes and Grafman 2010) and DA transmission, as this process has been shown to be affected in Parkinson’s patients and DA treatments in them (Salgado-Pineda et al. 2005; Sprengelmeyer et al. 2003).

We have recently shown that, in the visual preference paradigm test, macaques exhibited preferred attention to social visual stimuli (monkey faces with and without affective valences) than nonsocial stimuli (e.g., landscapes, objects) (Yamaguchi et al. 2017a). Administration of both DA D1 and D2 receptor antagonists decreased attention preference to social stimuli. However, D1, but not D2, antagonist increased attention to nonsocial stimuli at the same time, suggesting that D1 signaling maintains the balance of visual attention to social vs. nonsocial cues, whereas D2 signaling is more specifically involved in social information processing. However, the underlying neural mechanisms of such DA-dependent processing of visual social stimuli in primate species have still remained elusive.

Near-infrared spectroscopy (NIRS) has been utilized as a non-invasive brain activity measurement technique in humans, because of its relative easiness of use and safety. NIRS measures oxygenation (oxy-Hb) and deoxygenation (deoxy-Hb) of blood hemoglobins, which are correlated with cortical activity (Hoshi 2003; Madsen and Secher 1999). On the other hand, application of this technique in animals has barely been attempted. We have developed the method to conduct NIRS in non-human primates, unveiling its usefulness to investigate neural activity associated with categorization of visual stimuli in the PFC (Lee et al. 2017). Application of the technique such as NIRS in animals enables direct comparison with humans, and thereby further investigations on the neural mechanisms that mediate social information processing in non-human primates using NIRS would be a promising venue for understanding of how the neural mechanisms have evolved in primate species.

In this study, to elucidate the mechanisms of DA-dependent social information processing, we investigated PFC activities associated with social vs. nonsocial visual stimulus processing and the effects of pharmacological manipulations of DA transmission on them in Japanese macaques using NIRS. We hypothesized that oxy- and deoxy-Hb responses that were distinct between social and nonsocial visual stimuli were observed in the PFC of macaques. Moreover, these Hb changes associated with social stimuli were modulated by D1 and D2 antagonist administration.

## Materials and methods

### Subjects

All experiments were conducted in accordance with the *Science Council of Japan Guidelines for Proper Conduct of Animal Experiments* and approved by the Kyoto University Primate Research Institute Animal Experiment Committee. Two female Japanese macaques aged at 6 years old were used in this study. These monkeys were housed individually, and foods and water were available ad libitum throughout the experiments. They were trained to accustom to sit on a monkey chair, and stay quiet for up to 30 min per day.

### NIRS

**Fig. 1:**
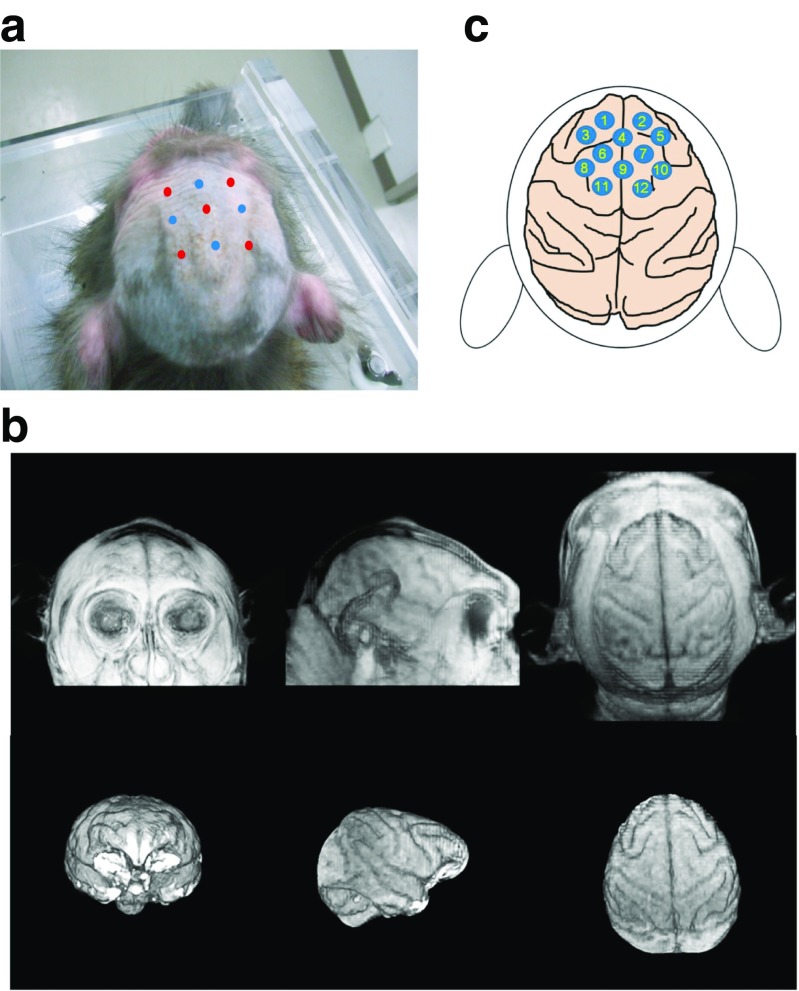
NIRS recordings in the PFC of macaques. **a** A photograph showing NIRS probe locations, with red and blue circles indicating emitters and detectors. **b** MRI scans showing the macaque brain and skull at coronal, sagittal, and horizontal views. **c** A schematic diagram illustrating recording sites (R1-R12) spanning over the PFC network

The setup (Hitachi ETG-100), which is originally developed for human use, was applied for recordings in primates. Details of NIRS recordings have been reported in our previous study (Lee et al. 2017). Nine probes allowing 12 points of measurements with the distance between an emitter and a detector at 1.5 cm were attached to the macaque skulls (Fig. 1a–c). Oxygenated (oxy-Hb), deoxygenated (deoxy-Hb), and total hemoglobin concentrations were recorded in the PFC regions, spanning over Brodman’s areas F2, F3, F6, F7, 8b, 9 l, 10, and 46d (Fig. 1a–c). Monkeys were first trained not to move in the monkey chair, and attachment of NIRS probes, along with maintaining their gazes on the LCD monitor for a minimum of 5 min, with intermittent rewards (drops of apple juice) during this period. Once the subjects were able to keep focusing on the LCD monitor, visual stimuli were presented. During NIRS recordings, we continuously monitored movements of monkeys with the CCD camera. In addition, elimination of potential motion artifacts was also achieved by a high pass filter at the frequency of 0.01 Hz in off-line analysis.

### Visual stimuli

Visual stimuli were presented to the macaques while they were gazing at the LCD monitors. Visual stimuli used in this study were similar to those we had used in our previous study (Yamaguchi et al. 2017a). The stimuli were divided into three categories. One was nonsocial images (NOS), such as trees, animals other than primates, foods (e.g., potato, apple), flowers, and landscapes. The other two categories were social images, both of which were macaque faces; one category was without emotional expressions (neutral faces; NUT), whereas the other category was with threatening facial expressions with teeth (emotional faces; EMT). All images were obtained from the internet (and thereby copy-protected), and adjusted to be approximately equal size. These images were semi-randomly presented on the LCD monitor that was placed approximately 50 cm apart from the subject’s eyes. While visual stimuli were presented, the room light was off, and experimenters controlled the equipment outside from the room. Whether the subjects gazed on the images was continuously monitored with the CCD camera. A single image was presented on the LCD screen in each trial. Each category of NOS, NUT, and EMT consisted of a set of 10 different images, such that a total of 30 different images was used in each subject. First, presentation of each of NOS, NUT, and EMT images were repeated twice in the same animals (thus, 20 image presentations per category per subject) in the control condition, ensuring that oxy-Hb and deoxy-Hb changes were relatively consistent at first and second presentations of the same images. Then, randomly selected 5 out of 10 images from each of NOS, NUT, and EMT categories (thus, 5 image presentations per category per subject) was tested for each of the D1 and D2 antagonist administration. Each trial consisted of 10 s of the gaze fixing stimulus (a red circle in the center of the LCD screen) followed by 30 s of image presentation. Inter-trial intervals were arbitrarily set for 20 to 60 s.

### Drug administration

The effects of DA D1 and D2 receptor antagonists on oxy-Hb and deoxy-Hb responses to visual stimuli were examined. The D1 antagonist SCH23390 (SCH) was dissolved in 3.0 ml of 0.9% saline, and given to the subjects at the dose of 0.5 mg/kg (i.m.). The D2 antagonist sulpiride (SUL) was dissolved in a drop of 1 N HCl, and diluted with 0.9% saline to be a final volume of 6.0 ml, and given subcutaneously to the subjects at the dose of 4.5 mg/kg. The equivalent volume of saline (SAL) was given as a control treatment. The doses of drugs were determined based on our previous macaque studies (Yamaguchi et al. 2017a, b, c), in which we tested the D1 and D2 antagonists for the visual attention task to social vs. non-social images as well as freely moving macaques in social groups. In particular, the doses of SCH23390 and sulpiride used in this study was not only previously found effective to alter various behavior of the drug-administered macaques and visual attention to social vs. non-social images, but particularly importantly, macaques were still able to sustain gazing to the images for a fixed duration during NIRS recordings. Time points for assessment of the drug effects after administration was determined based on the half-life of the drugs. Given the short half-life of SCH23390 (Kilts et al. 1985), NIRS recordings were started 5 min after drug administration, and completed less than 30 min after drug administration on each day. In contrast, sulpride exhibits a substantially longer half-life than SCH23390 (Wiesel et al. 1980), NIRS recordings were started 3 h after drug administration, and completed less than 30 min after drug administration on each day.

### Data analysis

**Fig. 2:**
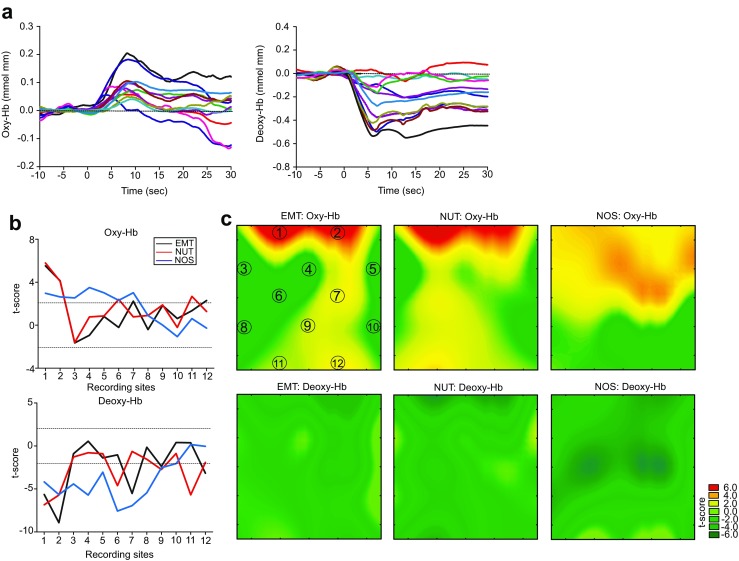
Oxy-Hb and deoxy-Hb changes in the PFC associated with social vs. nonsocial visual stimuli. **a** Representative examples of oxy-Hb and deoxy-Hb responses to presentation of a visual stimulus. Oxy-Hb and deoxy-Hb responses at each recording site are illustrated with different colors. **b** Graphs showing t-scores for oxy-Hb and deoxy-Hb changes at each recording site with EMT, NUT, and NOS stimuli. Above or below the dashed lines indicate statistically significant oxy- and deoxy-Hb changes compared to the baselines with one-sample *t* test. **c** Statistical parametric maps of the *t*-scores, illustrating distinct patterns of oxy-Hb and deoxy-Hb changes between EMT, NUT, and NOS stimuli. The circled numbers correspond to those in Fig. 1c

Since prolonged changes of oxy-Hb and deoxy-Hb concentrations were observed upon visual stimuli were observed (Fig. 2a), the area under the curve (AUC), i.e., summations of oxy-Hb and deoxy-Hb changes over the time, were calculated at each recording site for each image, and subsequent data analysis were conducted with the AUC. Data collection and statistical analyses were conducted by investigators who were not blinded to the experimental conditions. No data points were removed from statistical analysis. Sample sizes were not predetermined by statistical methods. All data analyses were conducted off-line. Binary data of the NIRS recordings were generated and used for statistical analyses using Origin Pro ver9.0 and Statistica ver7.0 software. A probability value of *p* < 0.05 was considered to indicate statistical significance.

## Results

### Oxy-/deoxy-Hb responses to social vs. nonsocial stimuli in the PFC

NIRS recordings with social and nonsocial visual stimulus presentation were conducted in two Japanese macaques. Presentation of social (macaque faces with (EMT) and without (NUT) affective valences) and nonsocial (NOS) visual stimuli evoked oxy-Hb and deoxy-Hb responses in the PFC regions (Fig. 2a). The AUC of oxy-Hb and deoxy-Hb responses to these visual stimuli were calculated for subsequent data analysis.

When EMT and NUT stimuli were presented, rigorous and focused oxy-Hb increases and deoxy-Hb decreases were observed in the most anterior part of the left and right PFC, which corresponded to the Brodman’s area 9 l and 10 (Fig. 2b, c). In contrast, more weak and broad increases and decreases of oxy-Hb and deoxy-Hb, respectively, were observed in the anterior half of PFC regions in response to NOS stimuli (Fig. 2b, c). In addition, a difference between EMT and NUT stimuli were also noticed in oxy-Hb increases and deoxy-Hb decreases, with EMT stimuli activating the right PFC, which corresponded to the Brodman’s area F6 and F7, whereas NUT stimuli activated the left PFC (Fig. 2b, c).

**Fig. 3:**
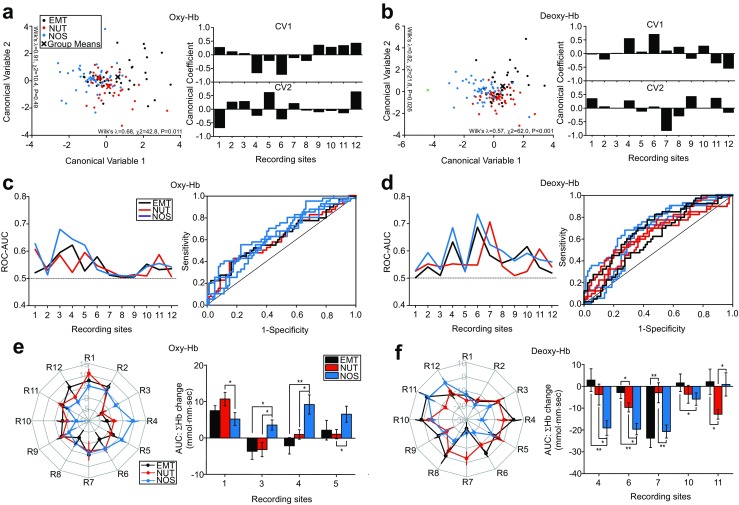
Oxy-Hb and deoxy-Hb responses to social and nonsocial visual stimulus presentation with SAL treatments. **a** Graphs showing discriminant analysis for oxy-Hb concentrations. **b** Graphs similar to (**a**), but showing for deoxy-Hb concentrations. **c** Receiver operating curve (ROC) analysis for oxy-Hb concentrations with one category of stimuli against the other two stimulus categories. The left graph shows AUC of ROC on each site of NIRS recordings for one category of stimuli over the other two categories. The right graph illustrates ROC for the recording sites where significant difference were observed for one category of stimuli over the other two categories. **d** Graphs similar to (**c**), but showing for deoxy-Hb concentrations. **e** A radar chart showing oxy-Hb changes at all recording sites (left) and a bar graph showing the recording sites where significantly different changes between stimuli were observed (right). *p < 0.05, **p < 0.01. Error bars indicate s.e.m. **f** Graphs similar to (**e**), but showing deoxy-Hb

To further elucidate how social and nonsocial visual stimuli evoked distinct patterns of oxy-Hb and deoxy-Hb changes in the PFC, linear discriminant analysis (Fisher 1936) was conducted by maximizing the ratio of between-visual stimulus category (EMT, NUT, and NOS) variance to the within-category variance in oxy-Hb and deoxy-Hb changes. In oxy-Hb changes, statistically significant wilk’s lamda, which indicated difference between the means of visual stimulus categories on a combination of dependent variables, was observed for the first canonical variable (Fig. 3a). High canonical coefficients were noticed in the recording sites 4 and 6 in the first canonical variable, and the recordings sites 1, 5, and 12 in the second canonical variable, indicating that oxy-Hb changes in these cortical areas may be involved in distinct processing of EMT vs. NUT vs. NOS stimuli (Fig. 3a). Similarly, in deoxy-Hb changes, statistically significant wilk’s lamda was observed for the first and second canonical variables (Fig. 3b). High canonical coefficients were noticed in the recording sites 4 and 6 in the first canonical variable, and the recording site 7 in the second canonical variable (Fig. 3b).

Receiver operating curve (ROC) analysis (Zweig and Campbell 1993) was also conducted to examine whether oxy-Hb or deoxy-Hb change in a specific recording site for one category of visual stimuli could distinguish over those for the other two categories of visual stimuli. In oxy-Hb, a statistically significant difference was found in response to EMT stimuli over NUT/NOS stimuli in the recording site 4 (Fig. 3c; Suppl. Table S1). Similarly, significant differences were found in response to NUT stimuli over EMT/NOS stimuli in the recording site 1, and in response to NOS stimuli over EMT/NUT stimuli in the recording sites 1, 3, 4, and 5 (Fig. 3c; Suppl. Table S1). In deoxy-Hb, statistically significant differences were found in response to EMT stimuli over NUT/NOS stimuli in the recording sites 4, 6, and 10, in response to NUT stimuli over EMT/NOS stimuli in the recording sites 7 and 11, and in response to NOS stimuli over EMT/NUT stimuli in the recordings sites 4, 6, and 7, respectively (Fig. 3d; Suppl. Table S1).

One-way ANOVA with post hoc Bonferroni test for oxy-Hb and deoxy-Hb changes on each recording site was conducted to further confirm distinct patterns of Hb responses to different visual stimulus categories. This statistical analysis unveiled significant differences for oxy-Hb changes between stimulus categories in the recording sites 1, 3, 4, and 5 (Fig. 3e; Suppl. Table S2), and deoxy-Hb changes in the recording sites 4, 6, 7, 10, and 11 (Fig. 3f; Suppl. Table S2).

Collectively, these results suggest that several PFC regions such as the recording sites 1, 4, 5, 6, which covers the Brodman’s area 9 l and 10 in the left hemisphere, 46d in the right hemisphere, and F6, F7, 8b in both hemispheres, exhibit distinct patterns of oxy-Hb and deoxy-Hb responses to EMT vs. NUT vs. NOS stimuli, and therefore may be involved in social vs. nonsocial information processing.

### Functional connectivity associated with social vs. nonsocial information processing

Whether distinct patterns of social vs. nonsocial information processing might also be detected as neural network activities within the PFC regions, we also investigated functional connectivity based on oxy-Hb and deoxy-Hb changes at each recording site with social and nonsocial stimulus presentation.

**Fig. 4:**
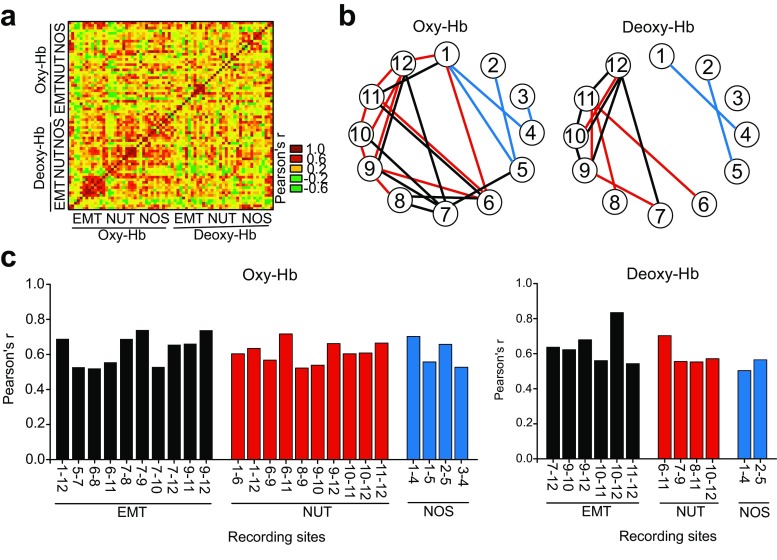
Functional connectivity in the PFC network based on oxy-Hb and deoxy-Hb changes with SAL treatments. **a** A correlation matrix with Pearson’s *r*. **b** Diagrams illustrating recording sites where statistically significant correlations with Bonferroni corrections are observed. **c** Graphs showing Pearson’s *r* for the pairs of recording sites where statistically significant correlations are observed

Multiple linear correlation analyses with Bonferroni corrections were conducted to examine connectivity between recording sites (Fig. 4a–c). Statistically significant positive correlations in response to EMT and NUT stimuli were observed between recording sites that were mostly located in the posterior PFC network (recording sites 6–12), whereas such correlations were observed mostly in the anterior PFC network (recording sites 1–5) in response to NOS stimuli (Fig. 4b, c; Suppl. Table S3). No statistically significant negative correlations with oxy-Hb and deoxy-Hb changes between recordings sites were observed in any category of visual stimuli.

These results suggest that social vs. nonsocial information processing may also involve distinct neural network activities in the PFC.

### DA modulation of oxy-/deoxy-Hb responses

To address how DA transmission might be involved in social vs. nonsocial information processing in the PFC, the effects of D1 and D2 receptor antagonist administration on oxy-Hb and deoxy-Hb changes was examined.

**Fig. 5:**
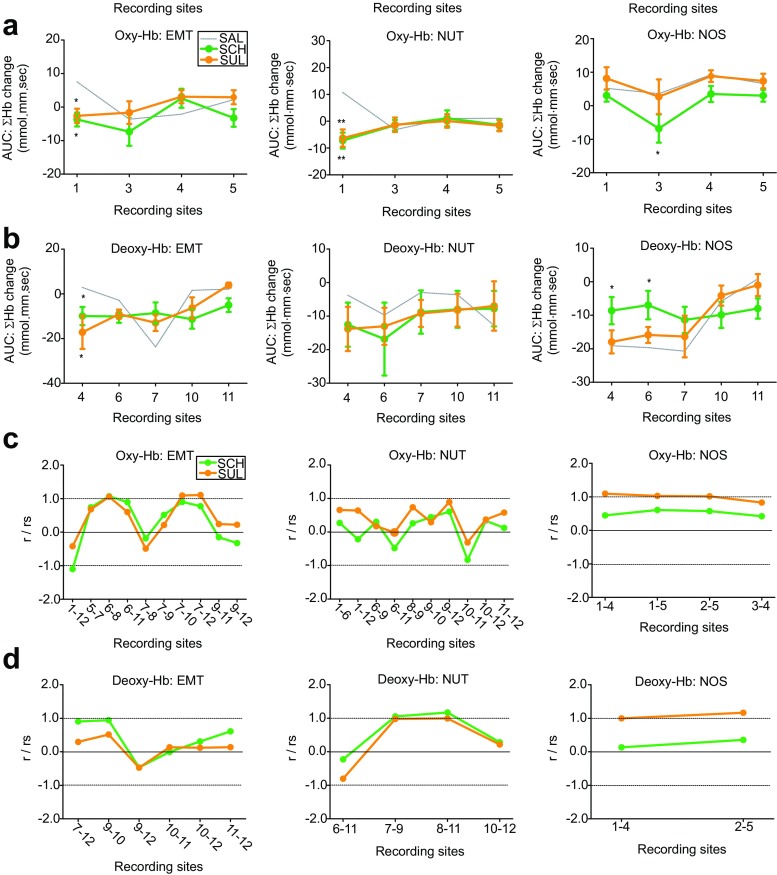
Oxy-Hb and deoxy-Hb responses and functional connectivity in the PFC to social and nonsocial visual stimulus presentation with DA antagonist treatments. **a** Graphs showing oxy-Hb changes with SAL (dashed, gray lines) and DA antagonists at the recording sites where significant differences were observed between stimulus categories with SAL treatments. *p < 0.05, **p < 0.01, relative to the SAL. **b** Graphs similar to (**a**), but showing for deoxy-Hb. **c** Graphs showing the ratios of Pearson’s *r* in oxy-Hb changes with DA antagonist treatments over those with SAL treatments (*r*_s_) for the pairs of recording sites where statistically significant correlations were observed with SAL treatments. The ratio of *r*/*r*_s_ = 1.0 indicates no alteration of correlation by drug administration relative to SAL, *r*/*r*_s_ = 0 indicates no correlation between recording sites with drug administration, and negative values on *r*/*r*_s_ indicate that positive correlation with SAL turns to negative correlation with drug administration. **d** Graphs similar to (**c**), but showing for deoxy-Hb

Using Wilcoxon signed-rank test, oxy-Hb and deoxy-Hb responses to EMT vs. NUT vs. NOS stimuli with SCH and SUL administration were compared with those with SAL administration at recording sites where significant difference between categories of visual stimuli were observed with SAL administration (as shown in Fig. 3e, f). In oxy-Hb changes, the most noticeable effects of DA antagonists were, first, that statistically significant alterations in response to EMT and NUT, but not NOS, stimuli were observed in the recording site 1 with both SCH and SUL administration (Fig. 5a; Suppl. Table S4); and second, that significant alterations in response to NOS stimuli were observed in the recording site 3 with SCH, but not SUL, administration (Fig. 5a; Suppl. Table S4). In deoxy-Hb changes, significant alterations in response to NOS stimuli were also observed in recording sites 4 and 6 with SCH, but none with SUL, administration (Fig. 5b; Suppl. Table S4).

Collectively, these results suggest that D1 receptor signaling is involved in both social and nonsocial information processing, whereas D2 receptor signaling is involved in primarily social, but not nonsocial, information processing in the PFC regions.

### DA modulation of functional connectivity

DA modulation of functional connectivity was further examined. We examined the effects of D1 and D2 antagonists on the linear correlations, by taking the ratios of linear correlations with drug administration (Pearson’s *r*) over those with SAL (*r*_s_), between the recording sites in which significant correlations were observed with SAL administration (as shown in Fig. 4c). Some, but not all correlations were affected by SCH and SUL administration (expressed as the ratio *r*/*r*_s_ lower or higher than 1.0; Fig. 5c, d). In particular, alterations of oxy-Hb and deoxy-Hb correlations associated with EMT and NUT stimuli were similar between SCH and SUL administration at each recording site, such that functional connectivity that were affected by SCH were similarly affected by SUL, whereas functional connectivity not affected by either one of drugs were also not affected by the other drug (Fig. 5c). In contrast, although functional connectivity with NOS stimuli were altered by SCH administration, SUL administration did not affect these correlations (Fig. 5d).

These results suggest that both D1 and D2 receptor signaling may be involved in social information processing, whereas D1, but not D2, receptor signaling may be also involved in nonsocial information processing, based on functional connectivity in the PFC network.

## Discussion

In this study, we have shown that distinct patterns of oxy-Hb and deoxy-Hb responses in the PFC were observed between social and nonsocial visual stimuli. Moreover, presence or absence of affective valences in the social stimuli resulted in activation of the PFC network in the opposite hemisphere. Functional connectivity within the PFC network expressed as correlated oxy-Hb and deoxy-Hb changes were observed in more anterior parts with nonsocial stimuli, whereas such functional connectivity was prominent in more posterior parts with social stimuli. In addition, we also found that both D1 and D2 antagonists modulated oxy-Hb and deoxy-Hb changes and functional connectivity associated with social stimuli in similar manners. In contrast, D1, but not D2, antagonist modulated these responses with nonsocial stimuli.

Human studies have shown important roles of the PFC in social cognition. In particular, social cognition appears to be divided into implicit and explicit ones, and the PFC is involved in explicit social cognitive processes (Cunningham and Zelazo 2007; Forbes and Grafman 2010). Such explicit social cognition includes recognition of self and others, as well as recognition of mental states of others, i.e., theory of mind. Accumulating evidence suggests that the medial PFC in the right hemisphere play critical roles in such explicit social cognition as recognition of self (Keenan et al. 2000; Uddin et al. 2005). Moreover, subjects with psychiatric disorders such as autism spectrum disorder (ASD) and schizophrenia exhibit deficits in explicit social cognition and decreased activation of the medial PFC (Castelli et al. 2002; Mohnke et al. 2016). ASD subjects are also found to exhibit higher activation of multiple PFC regions including the dorsolateral PFC than normal subjects when they are attending to faces (Herrington et al. 2015). Our current study in macaques is partly consistent with those human studies, as we found that presentation of macaque faces with emotional expressions primarily activates the medial PFC of the right hemisphere, whereas macaque faces without affective valences activates the mPFC of the left hemisphere. The reason of such medial PFC activation in the opposite hemisphere depending on presence or absence of emotional expressions in faces still remains unclear. However, human studies have also shown lateralized cortical activation by emotional faces, with emotional processing more strongly involved in the cortex of right than left hemisphere (Indersmitten and Gur 2003; Killgore and Yurgelun-Todd 2007).

We found that anterior PFC sites showed activation to social stimuli, and rather posterior sites showed activation to non-social stimuli. However, functional connectivity analyses showed the opposite (posterior connectivity with social stimuli and anterior connectivity with non-social stimuli). The reason for such discrepancy between Hb changes and functional connectivity has remained unknown. However, similar to these observations, in a functional magnetic imaging study, non-overlapping regional patterns of blood oxygenation level-dependent signals and functional connectivity, and even negative correlations between them during a continuous visual attention task has also been reported (Tomasi et al. 2014).

Although the roles of mesocortical DA transmission in the PFC for cognitive functions in nonsocial domains have been extensively studies (Arnsten 1997; Robbins 2000; Seamans and Yang 2004), its roles on social cognition have remained elusive. That DA is involved in recognition of emotional expressions of faces has been reported in studies with Parkinson’s patients under therapeutic treatments with L-dopa (Salgado-Pineda et al. 2005; Sprengelmeyer et al. 2003). We have also recently shown that DA regulates visual attention preference to social over nonsocial stimuli, but through distinct mechanisms with D1 and D2 receptor signaling (Yamaguchi et al. 2017a). Thus, although D1 receptor signaling appears to balance attention preference between social and nonsocial cues, D2 receptor signaling appears to be more specifically involved in social processing. The findings in the current study are consistent with those previous findings, as D1 antagonist administration affected oxy-Hb and deoxy-Hb changes associated with both social and nonsocial cues, whereas D2 antagonist administration affected oxy-Hb and deoxy-Hb changes associated with social, but not nonsocial cues. Thus, the current study suggests that these previous observations may partly be mediated through mesocortical DA transmission in the PFC.

NIRS has been developed to measure cortical activities in humans, and its application to animal models have been limited. However, application of the technique such as NIRS in animal models enables direct comparison of investigations between animals and humans, and thereby yield great advantages in translational research. In particular, a study such as ours that combines NIRS recordings with pharmacological manipulations in non-human primates could be a powerful approach for understanding of the biological mechanisms of psychiatric disorders and developments of their therapeutic treatments.

## Electronic supplementary material

ESM 1 (PDF 39 kb)
