# Supplementary material for: Dopamine-dependent social information processing in non-human primates
Source: Psychopharmacology (Berl). 2018 Jan 14;235(4):1141–9. doi: 10.1007/s00213-018-4831-x (PMC5869898; doi:10.1007/s00213-018-4831-x)
Supplement: Supplementary file 1 — (PDF 39 kb) [file 213_2018_4831_MOESM1_ESM.pdf]

Supplemental Material

Dopamine-dependent Social Information Processing in Non-human Primates

Young-A Lee[1], Sarah Lionnet[2], Akemi Kato[3], & Yukiori Goto[3]

[1]*Department of Food Science and Nutrition, Daegu Catholic University, Gyeongsan, Gyeongbuk, 38430, South Korea*

[2] *Ecole Nationale Veterinaire de Toulouse, Toulouse, 31076, France*

[3]*Primate Research Institute, Kyoto University, Inuyama, Aichi, 484-8506, Japan*

E-mail address of the corresponding author: goto.yukiori.5c@kyoto-u.ac.jp

Supplemental Table S1

Supplemental Table S2

Supplemental Table S3

Supplemental Table S4

Supplementary Table S1. A summary of p-values in ROC analysis.

Oxy-Hb

	EMT	***NUT***	***NOS***
Recording site 1	0.70916	0.04765*	0.02313*
2	0.44234	0.59304	0.81513
3	0.09057	0.12856	0.00132*
4	0.0295*	0.69676	0.01028*
5	0.62815	0.08952	0.02909*
6	0.15735	0.40369	0.56261
7	0.82812	0.62421	0.78502
8	0.94231	0.91576	0.85861
9	0.92018	0.80219	0.88052
10	0.35253	0.87174	0.44234
11	0.56637	0.12038	0.32719
12	0.52566	0.90693	0.45232

Deoxy-Hb

	EMT	***NUT***	***NOS***
Recording site 1	0.97779	0.64803	0.62815
2	0.46583	0.35253	0.09712
3	0.86298	0.45568	0.56637
4	0.01798*	0.35253	0.00098*
5	0.65204	0.38515	0.67629
6	0.00089*	0.39127	0.00029*
7	0.13717	0.00024*	0.02868*
8	0.32172	0.43248	0.07575
9	0.19651	0.86736	0.26078
10	0.03889*	0.66009	0.10404
11	0.48303	0.04620*	0.22703
12	0.73836	0.46583	0.28765

*Statistically significant difference

Supplementary Table S2. A summary of statistical analysis for oxy-Hb and deoxy-Hb changes with one-way ANOVA.

Oxy-Hb

	$F_{2,117}$	*p*	*Post-hoc*		
			EMTvs.NUT	*EMTvs.NOS*	*NUTvs.NOS*
Recording site 1	3.15480	0.04630*	0.45812	0.86980	0.04114*
2	0.33570	0.71551	1.00000	1.00000	1.00000
3	4.55414	0.01243*	1.00000	0.02400*	0.03927*
4	7.51280	0.00085*	0.91841	0.00083*	0.02239*
5	3.21624	0.04368*	0.27816	1.00000	0.04353*
6	1.33794	0.26637	0.45314	0.50501	1.00000
7	0.09347	0.91083	1.00000	1.00000	1.00000
8	0.45732	0.63410	1.00000	1.00000	1.00000
9	0.62616	0.53642	1.00000	0.89366	1.00000
10	0.94940	0.38993	1.00000	0.53599	1.00000
11	0.78649	0.45783	1.00000	1.00000	0.67303
12	1.84933	0.16191	1.00000	0.44481	0.21556

Deoxy-Hb

	$F_{2,117}$	*p*	*Post-hoc*		
			EMTvs.NUT	*EMTvs.NOS*	*NUTvs.NOS*
Recording site 1	0.75564	0.47199	0.95676	1.00000	0.79685
2	0.61080	0.54462	1.00000	1.00000	0.86095
3	1.14422	0.32201	1.00000	0.62720	0.53647
4	6.22959	0.00269*	0.96059	0.00249*	0.04948*
5	0.29488	0.74517	1.00000	1.00000	1.00000
6	12.0790	0.00002*	0.04953*	0.00001*	0.04339*
7	7.82300	0.00065*	0.00113*	1.00000	0.00669*
8	2.03032	0.13589	1.00000	0.15075	0.56692
9	0.60520	0.54769	0.84207	1.00000	1.00000
10	3.06275	0.05053	1.00000	0.04961*	0.32751
11	4.12334	0.01860*	0.03780*	1.00000	0.04893*
12	1.05944	0.34995	0.96748	0.47647	1.00000

*Statistically significant difference

Supplementary Table S3. A summary of p-values in multiple linear correlation analyses between pairs of recording sites.

Oxy-Hb

EMT		***NUT***		***NOS***	
Recording pairs	***p***	***Recording pairs***	***p***	***Recording pairs***	***p***
1-12	<0.0001	*1-6*	<0.0001	*1-4*	<0.0001
5-7	0.0005	*1-12*	<0.0001	*1-5*	0.0002
6-8	0.0006	*6-9*	0.0001	*2-5*	<0.0001
6-11	0.0002	*6-11*	<0.0001	*3-4*	0.0005
7-9	<0.0001	*8-9*	0.0006		
7-10	0.0005	*9-10*	0.0003		
7-12	<0.0001	*9-12*	<0.0001		
9-11	<0.0001	*10-11*	<0.0001		
9-12	<0.0001	*10-12*	<0.0001		
		11-12	<0.0001		

Deoxy-Hb

EMT		***NUT***		***NOS***	
Recording pairs	***p***	***Recording pairs***	***p***	***Recording pairs***	***p***
7-12	<0.0001	*6-11*	<0.0001	*1-4*	0.0006
9-10	<0.0001	*7-9*	0.0002	*2-5*	0.0001
9-12	<0.0001	*8-11*	0.0002		
10-11	0.0002	*10-12*	0.0001		
10-12	<0.0001				
11-12	0.0003				

*Statistical significance at p=0.0007 or lower with Bonferroni correction

Supplementary Table S4. A summary of statistical analysis for D1 and D2 antagonist effects on oxy-Hb and deoxy-Hb changes with Wilcoxon signed-rank test.

Oxy-Hb

	EMT		***NUT***		***NOS***	
SCH vs. SAL	***Z***	***p***	***Z***	***p***	***Z***	***p***
Recording site 1	2.213594	0.026857*	2.846050	0.004427*	0.316228	0.751830
3	1.581139	0.113846	-0.316228	0.751830	2.213594	0.026857*
4	-0.948683	0.342782	0.316228	0.751830	1.581139	0.113846
5	1.581139	0.113846	0.316228	0.751830	1.581139	0.113846
SUL vs. SAL						
Recording site 1	2.213594	0.026857*	2.846050	0.004427*	-0.948683	0.342782
3	-0.316228	0.751830	-0.316228	0.751830	0.316228	0.751830
4	-0.948683	0.342782	0.316228	0.751830	0.316228	0.751830
5	-0.316228	0.751830	0.316228	0.751830	-0.316228	0.751830

Deoxy-Hb

	EMT		***NUT***		***NOS***	
SCH vs. SAL	***Z***	***p***	***Z***	***p***	***Z***	***p***
Recording site 4	2.213594	0.026857*	0.316228	0.751830	-2.213594	0.026857*
6	0.948683	0.342782	0.316228	0.751830	-2.213594	0.026857*
7	-0.948683	0.342782	0.316228	0.751830	-0.948683	0.342782
10	1.581139	0.113846	0.316228	0.751830	0.948683	0.342782
11	1.581139	0.113846	-0.316228	0.751830	1.581139	0.113846
SUL vs. SAL						
Recording site 4	2.213594	0.026857*	0.316228	0.751830	-0.316228	0.751830
6	0.316228	0.751830	0.316228	0.751830	-0.948683	0.342782
7	-0.948683	0.342782	0.316228	0.751830	-0.316228	0.751830
10	0.948683	0.342782	0.316228	0.751830	-0.316228	0.751830
11	0.316228	0.751830	-0.316228	0.751830	0.316228	0.751830

*Statistically significant difference
